# Numerical analyses of intestinal microbiota by data mining

**DOI:** 10.3164/jcbn.17-84

**Published:** 2018-01-11

**Authors:** Toshio Kobayashi, Akira Andoh

**Affiliations:** 1 Miyagi University, 2-2-1 Hatatate, Taihaku-ku, Sendai-Shi, Miyagi 982-0215, Japan; 2 Department of Medicine, Shiga University of Medical Science, Seta-Tsukinowa, Otsu, Shiga 520-2192, Japan

**Keywords:** intestinal microbiota, terminal restriction fragment length polymorphism, operational taxonomic unit, data mining, decision tree

## Abstract

The human intestinal microbiota has a close relationship with health control and causes of diseases, and a vast number of scientific papers on this topic have been published recently. Some progress has been made in identifying the causes or species of related microbiota, and successful results of data mining are reviewed here. Humans who are targets of a disease have their own individual characteristics, including various types of noise because of their individual life style and history. The quantitatively dominant bacterial species are not always deeply connected with a target disease. Instead of conventional simple comparisons of the statistical record, here the Gini-coefficient (i.e., evaluation of the uniformity of a group) was applied to minimize the effects of various types of noise in the data. A series of results were reviewed comparatively for normal daily life, disease and technical aspects of data mining. Some representative cases (i.e., heavy smokers, Crohn’s disease, coronary artery disease and prediction accuracy of diagnosis) are discussed in detail. In conclusion, data mining is useful for general diagnostic applications with reasonable cost and reproducibility.

## Introduction

It is well known that the human intestinal microbiota has a close relationship with health control and causes of diseases. More than 10 reviews on this topic were published between March and May 2017, with a focus on the species of microbiota that are related to certain characteristics of diseases. Thus, the volume of reports and new findings is growing every month. Unfortunately, the methods of statistical analysis for reducing or identifying related microbiota have remained the same, and ignore the characteristics of obtained human data, which contain various individual types of noise because of differing life styles and history.

The following reviews of intestinal microbiota and their influence on daily life and diseases can be highlighted. Singh *et al.*^(1)^ reviewed the broad relationship of food intake in altering gut microbiota, and included numerous tables concerning the intake of protein, fat, carbohydrate, as well as obesity and some diseases. An example is shown here in Table 1. The relationship between microbiota and the kinds of protein is indicated qualitatively with arrows. Witte *et al.*^(2)^ reviewed reports on autoimmune diseases and metabolic disorders and discussed fecal microbiome transfer. Seganfredo *et al.*^(3)^ reviewed the association of microbiota with being overweight or obese and discussed the imbalances of gut microbiota and prebiotics. Clark *et al.*^(4)^ described the unclear etiology of acne vulgaris and proposed a gut–skin axis, which has been rarely discussed. Tang *et al.*^(5)^ reviewed various diseases, including cardiovascular diseases, dysbiosis, atherosclerosis, hypertension, heart failure, chronic kidney disease, obesity and type 2 diabetes, and discussed the potential of modulating intestinal microbial inhabitants as a novel therapeutic strategy. Kang and Cai^(6)^ reviewed hepatitis B and chronic liver disease, and discussed the forefront relationship between gut microbiota and hepatitis B virus-induced chronic liver disease and the prospects for therapy. Doulberis *et al.*^(7)^ reviewed nonalcoholic fatty liver disease, with a special focus on the role of gut microbiota, and noted that probiotics might have a positive role in their management. Armani *et al.*^(8)^ reviewed chronic kidney disease and argued that excessive amounts of uremic toxins are implicated in its progression as influenced by gut microbiota. The contributions of microbiota to pediatric liver disease, nonalcoholic steatohepatitis, and primary sclerosing cholangitis were reviewed by Leung and Yamada.^(9)^ These conditions differed in both severity and rate of progression depending on the patients. Carvalho *et al.*^(10)^ reviewed the dysfunctional interaction between the intestinal microbiota and the host immune system, known as dysbiosis, with gastrointestinal inflammatory diseases, inflammatory bowel diseases, and mucositis. Li *et al.*^(11)^ reviewed autism spectrum disorder, inflammatory bowel disease, and mood disorders, which suggest bidirectional interactions between the central nervous system and the gastrointestinal tract (brain–gut axis) and the role of the gut microbiota in the central nervous system. Zhang *et al.*^(12)^ described the gastrointestinal microbiome as it is related primarily to mechanisms of gastric carcinogenesis and gastric malignancies within the upper gastrointestinal tract. Miraghajani *et al.*^(13)^ reviewed 30 articles on the intestinal mucosa and microbiota, anti-inflammatory and immunomodulatory effects on endoplasmic reticulum stress, and the expression of genes involved in glucose homeostasis and insulin resistance. Haque and Haque^(14)^ described the significant association between microbiota and digestion, metabolism, and the immune system of its host. All these reviews are recent, and the range of target diseases reviewed has expanded widely and the related microbiota were compared qualitatively (as Table 1).^(1–14)^

On the premise of these associations, the aim of this review is to focus on a way to clearly identify the causes or species of microbiota related to a disease. Some examples of cases where successful results of quantitative identification have already been obtained are summarized below.

## Successful Results of Quantitative Identification

If some members of specific groups can be gathered, data on their intestinal microbiota can be collected from their feces; however, the following analyses of the numerical data will encounter a new challenge. To identify the characteristics of the microbiota existing in specific groups, conventional statistical methods to analyze such a complex relationship cannot clearly identify differences between these groups. In other words, it cannot be said that the differences in large groups always represent their differences. Mathematical methods that lead to identification of differences between groups more clearly than conventional statistics are required. Therefore, we have introduced data mining analysis (DM) to identify clearly the differences between target groups or diseases.

### Characteristics of DM

DM was originally developed to identify effectively social scientific data that include various types of noise. Similarly, regarding the intestinal microbiota in which we are interested, even if the subjects have the same disease, because their age, sex, lifestyle, etc., are different, the obtained data are not necessarily related to the same bacterial content. As the volume of data increases, the influence of such noise becomes greater, and so a specific method to distinguish microbiota that can characterize the groups becomes indispensable. Results of using DM as a practical method are shown below.

DM discriminates by using the Gini-coefficient, which compares and optimizes the difference of uniformity of each group. As a result, the most abundant bacterial species are not the characterizing bacteria. Accordingly, the seventh or twentieth most abundant species have been selected by their optimized Gini-coefficients, which are occasionally their most important characteristic. In addition, DM constructs the decision tree (Dt) that identifies all the groups to be compared step-by-step (https://www.ibm.com/us-en/marketplace/spss-decision-trees), divides them into several ‘nodes’, and shows easily the visual and quantitative configuration of the target group. All DM processing was conducted using IBM SPSS Clementine ver. 14 and 15.

### Overall results

Table 2 shows the overall results analyzed by DM for various targets. The targets of the DM analyses were divided into the three parts vertically, i.e., daily life, disease and technical aspects of DM. In Table 2, under ‘Results of DM-analyses’, the outcomes of individual analysis are indicated vertically. & indicates good identification of all the members of the target up to five steps of Dt. # indicates observation of a few errors at the same steps, which might have some another distinguishing item, such as sex differences or some other unknown items affecting the configuration of the microbiota.

Most of the bacterial analyses from fecal samples were performed by terminal restriction fragment length polymorphism (T-RFLP) with various restriction enzymes (REs), and a few were analyzed using 16S rRNA sequencing amplicon analysis (NGSA) and amino acid content in fasting blood. The gray cells of Table 2 are described in further detail below.

### Daily life

The boundary between daily life and disease is not clear, and we treated it here as shown in Table 2, namely, smoking and drinking habit belong to disease. The data for age and BMI are continuous numerical values,^(16)^ which differs from category values or nominal partitions, e.g., A or B, so the structures of obtained Dts were similar but rather different. The Dts were much more precise and complicated to exhibit. Moreover, continuous numerical values could not be correlated directly with DM estimation. However, a merit of using these continuous values was that we could easily divide the subjects at any point within cited area, so the 92 men were separated into a certain 2 parts, both by age (21–59 years old) and BMI (17.3–30.2), i.e., younger and older groups and skinny or fatty ones, and compared. Various dividing points with age and BMI were applied using four REs, i.e., *Alu*I, *Bsl*I, *Hae*III, *Msp*I, and obtained Dts by optimization of Gini-coefficient were all successful for precisely understanding the roles of certain OTUs. It became clear that some OTUs are closely related to age and BMI, and another OTUs are irrelevant to them.

The microbiota of 121 individuals who live in four Japanese cities hundreds of km apart, Chiba, Shiga, Hyogo, Fukuoka, shows that there are general and clear differences using two OTUs, *Hha*I, *Msp*I.^(18)^ Obtained Dt indicates to sort out the most subjects correctly, but does a few misidentifications. Considering the migration of urban dwellers, it seems natural that some false appear.

### Diseases

**Fig. 1:**
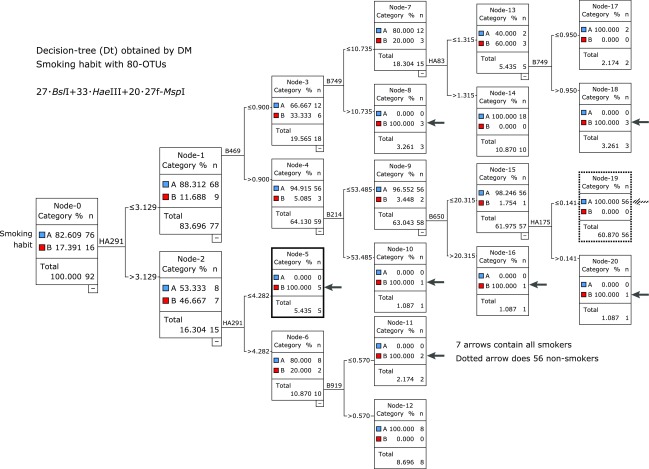
Smoking habit: Dt obtained by DM with 92 healthy men. Each square is called ‘node’. The left end node is called ‘Root-node’, which is the starting point of tree construction. Dt was growing toward right side. The marks, e.g., HA291, was the dividing OTU, of which numerical dividing points were shown. Each node showed its component of subjects. A: non-smokers, B: smokers; Reprinted with permission from Ref (15).

The focus of research on intestinal microbiota has been the investigation and diagnosis of various diseases. Among the notable targets here are smoking habits. The results analyzed by DM of 92 healthy men (aged 21–59 years, 16 present smokers) are shown in Fig. 1. The Dt identified A: 76 nonsmokers and B: 16 smokers. The most distinguishing OTU was HA291 (*Hae*III-291), and 92 subjects were divided into two subsets Node-1 and Node-2, and each subset was more homogeneous than the previous Node-0. Similarly, the identifying procedures occurred step-by-step from left to right, and the Dt was constructed (Fig. 1). Finally, all subjects were clearly identified with 8 OTUs; in other words, the other 72 (= 80 – 8) OTUs were not related to smoking. However, considering Node-5 in Fig. 1 (bold line square), it is noteworthy that all four heavy smokers (≥20 cigarettes/day) among the 16 gathered in Node-5, with HA291 used twice. It is also noteworthy that most nonsmokers, 56 (74% = 56/76) gathered at the rightmost Node-19 (bold dotted line square). We examined the ranking in descending order of abundance of OTUs, and HA291 was ranked 12th with 92 individuals. In contrast, when examining the same ranking in 16 smokers, HA291 was ranked 10th in 33 OTUs of *Hae*III. And DM analyses succeeded in including former smokers whose smoking cessation period was more than 1 month to 26 years.^(16)^

**Fig. 2:**
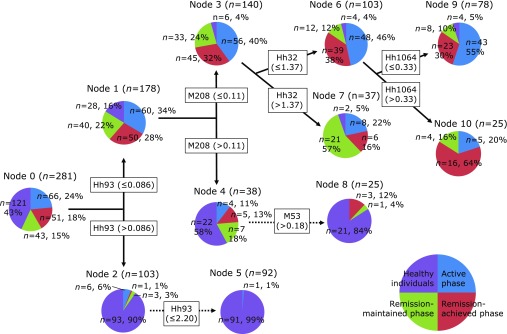
Dt results of ‘Crohn’s disease’. *Hha*I 93-bp OTU and *Msp*I 208-bp OTU are abbreviated as Hh93 and M208. The cut-off values are also calculated by Gini-coefficient. The details of the decision tree and the pathway indicate the species and quantities of OTUs. Reprinted with permission from Ref (19).

We also obtained other DM results. A Dt for Crohn’s disease is shown in Fig. 2. The transitions of microbiota in three phases, i.e., active phase (66 subjects), remission-maintained phase (51 subjects), and remission-achieved phase (43 subjects), were compared with healthy individuals (121 subjects) with *Hha*I and *Msp*I. Four components [nominal partitions (NP)] of the group of 281 subjects were identified with DM, and 5 OTUs (Hh93, M208, Hh32, M53, Hh1064) of 99 OTUs (42-*Hha*I + 57-*Msp*I) were active to distinguish these complicated cases. If we pay attention to the ‘healthy individuals’ in Fig. 2, we note a few identifying errors at the upper right with respect to Node-7, Node-8 and Node-9.

**Fig. 3:**
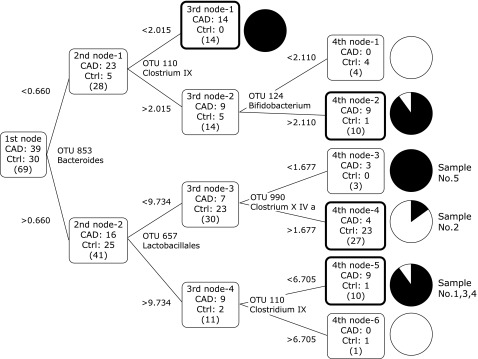
Dt results of ‘Coronary artery disease’ with *Bsl*I. Coronary artery disease, CAD; control, Ctrl. Dark areas in circle charts represent CAD. The cut-off values of each dividing steps are calculated with optimization of Gini-coefficient. Reprinted with permission from Ref (24).

For coronary artery disease (CAD), the results of the Dt are reported in Fig. 3. The 39 patients with CAD were compared with 30 ages- and sex-matched no-CAD controls (Ctrl) with *Bsl*I. Circles on the right side of this figure are terminal nodes, i.e., ends of dividing lines of the Dt, and the dark areas in the circles represent the patients with CAD. The misclassified patients (6/39, 15.4%) were observed at three nodes.

Furthermore, to test a diagnostic prediction using the same Dt, we included five new additional CAD patients. Four of them separated into the CAD node (4th Node-3 and 4th Node-5 in Fig. 3), and one belonged to the Ctrl node (4th Node-4), where four CAD patients were previously found. Then, there were five patterns of CAD pathology seen from the intestinal microbiota, and *Bsl*I-853 (*Bacteroides*), *Bsl*I-110 (*Clostridium* IX), and *Bsl*I-657 (*Lactobacillales*) were closely related to whether CAD develops or not for these subjects.

DM analyses are effective not only for analyses involving intestinal microbiota, but also for a variety of medical data, e.g., amino acid composition of fasting blood obtained in the early morning for identifying dementia. Various analyses were used and separate analyses by sex provide much better results than mixed sex data. With intestinal microbiota, Dt differences based on sex are observed with diabetes, i.e., type 1, type 2 and controls, namely, 3-NP.^(25)^

### Technical aspects of DM

Much effort has been made to increase the efficiency and accuracy of DM analyses. There are three different focal points for results: the selection of REs, prediction accuracy, and individual identification of subjects. These are described as follows.

The selection of REs is extremely important for obtaining a clear and successful Dt for certain targets with T-RFLP analyses of intestinal microbiota. When dealing with a new disease, it is necessary to apply as many REs as possible, e.g., three or more, to find the closely related OTUs; for example, HA291 for smokers in Fig. 1.^(15)^ Once a clear Dt is constructed, we can reduce the number of REs for the same disease according to the reasons for DM processing, i.e., predictive diagnosis of diseases or detailed pathology. The former requires fewer REs than the latter. In Table 2, we can see that the ‘most related OTUs’ of the vertical line, 516f-*Hae*III, 516f-*Bsl*I, 27f-*Msp*I, *Hha*I, and 27f-*Alu*I, are effective for DM analyses according to different targets, but the 35f series of REs are empirically ineffective at the present stage.

The most important characteristic of DM analysis is how accurately it is possible to identify the group of microbiotas related to targets. Considering the dependence on the unknown relationship between a target and microbiota data, much trial and error is required to determine effective DM processing. Here, we compared the prediction accuracy from the results obtained by using REs widely used to identify smokers and drinkers. Table 3 indicates how misidentifications by DM (i.e., false nodes) occur in Dt constructs. In Table 3, seven kinds of REs, including the 35f series of three REs, were applied to the same target, and the results (i.e., the ‘number of false identifications in 92 records’) are compared. Naturally, 0 is the best; the case of Fig. 1 (B+HA+M, for smoking) is not shown in Table 3. There was unknown compatibility between the target and REs; increasing the number of REs is a powerful way to obtain fewer false identifications. ‘Dilute, Conc., D2/C1, and $’ in Table 3 represent the fine features of errors and they are described precisely.^(17)^

Another feature of DM is that we can track the configuration of an individual from a large data group, which is extremely important in diagnosis of a certain patient. To track the changes of the microbiota before and after the treatments, the individual detailed dynamics are indispensable, and cannot be provided by conventional statistical methods of observing unified and global analyses. The stepwise application and optimization of the Gini- coefficient by DM enables these distinctive individual tracking procedures.

## Discussion

To date, DM analyses have not been widely applied in the field of medicine. Although many publications have similar titles to this paper, their content is different,^(26,27)^ especially for analyses of intestinal microbiota. However, since DM has many features, a growth in applications and practices is expected. A notable chapter entitled ‘All diseases begin in the gut’ by Collen appeared in the 2015 work *10% Human: How Your Body’s Microbes Hold the Key to Health and Happiness*.^(28)^ However, although research efforts are currently advancing, a step-by-step approach is desirable to accumulate a large volume of research data.

### Technical viewpoints

Here, we focused on the numerical analyses of the intestinal microbiota, and reviewed and compared a broad range of works. The DM method can be widely applied to two-dimensional numerical data with one restriction; that is, the number of horizontal information quantity terms (fields), e.g., species of OTU, must be almost the same or smaller than the number of vertical subject terms (records). It is not possible to analyze cases that have a vast number of informational terms for a small number of subjects, such as metagenomic data.

As shown in Table 2, microbiota was previously analyzed mostly by T-RFLP analyses with some REs. Recently, 16S rRNA sequencing amplicon analysis (NGSA) has been well developed and advanced research on microbiota is progressing rapidly. However, considering routine diagnoses of diseases for many individuals, T-RFLP analyses are more effective than NGSA in terms of the cost of fecal analyses and the reproducibility of the obtained numerical data. After the field of DM has progressed, the method will allow us to know importantly the pathological condition and chronological transition of a subject’s microbiota than to repeatedly confirm the already known qualitative names of bacteria. That is, DM will become applicable to general predictive diagnostic techniques, of which examples were cited in the later part of the section ‘Disease’ concerning CAD. For that purpose, it is necessary to increase the number of subjects and the cases of various diseases, which must be much greater than shown in Table 2. If the number of subjects is greatly increased, then we will encounter some new currently unknown challenges of DM processing. This will greatly help to alleviate the national medical expenses which have been increasing every year. While, most challenges will lead to the discovery of important factors that affect the precise mechanisms and activities of intestinal microbiota. As an example, sex differences have already been confirmed for dementia and diabetes; however, age of subjects,^(16,20)^ area of residence,^(18)^ and ethnicity are expected to affect microbiota. Although the actual pathological situations related to microbiota have begun to be clarified in part by DM, much remains to be done.

### Medical viewpoints

Inflammatory bowel diseases (IBD), including ulcerative colitis and Crohn’s disease, are chronic intestinal disorders of multifactorial etiology. Although the precise pathogenesis of IBD remains poorly understood, dysregulated host–microbial interactions are considered to play a role in initiating and perpetuating IBD. Particularly, an alteration of the diversity and composition of the gut microbiome (dysbiosis) rather than the presence of specific pathogens likely plays a critical role. As shown in Fig. 2, healthy individuals and patients with Crohn’s disease were clearly separated. Almost all healthy individuals were in Nodes 5 and 8, and patients with Crohn’s disease were ultimately located in Nodes 7, 9 and 10. Furthermore, Nodes 9 and 10 were mainly characterized by active phase and remission-achieved phase, respectively. Node 7 mainly comprised remission-maintained phase. Thus, DM analyses with T-RFLP data were useful for characterization of the gut microbial community at different phases of clinical activity of Crohn’s disease. This suggests an applicability of DM analysis to clinical data of the intestinal microbiota in various diseases.

How is it possible to identify intestinal microbiota species for many disease targets by DM analysis? Although still at a hypothetical stage, it is believed that “there are some waste products generated in the human body, which are polymer compounds and excluded from the intestinal tract. There are also unique intestinal bacteria related to the discharge of each waste product. Whether symbiotic anchoring of related bacterial groups in the body is a branch point of personal tolerance to many targets is not known now.” The explanations lie in the result in Fig. 1 for smoking habit. Compared with nonsmokers, certain waste is generated by smokers in accordance with their habit, and this waste causes symbiosis of bacterial species within the body that permanently discharge waste from the body.

Although still at an early stage of research, DM analysis appears to be promising as an important tool for analyses of intestinal microbiota.

## Figures and Tables

**Table 1:** Singh RK *et al.* “Effects of protein on gut microbiota”

	Microbial diversity	*Bifidobacteria*	*Lactobacilli*	*Bacterodes*	*Alistipes*	*Bilophila*	*Clostridia*	*Rosoburia*	*Eubacterium Rectale*
Animal protein	↑	↑↓		↑↓	↑	↑	↑	↓	↑↓
Whey protein extract	↑	↑	↑	↓			↓		
Pea protein extract	↑	↑	↑						

Arrow thickness corresponds to relative number of studies supporting the relationship; Reprinted with permission from Ref (1).

**Table 2:** List of the successful results by data-mining analysis

Target		Number of subjects			T-RFLP or NGSA	Results of DM-analyses	Most related OUT	References
		Target	Ref	Total				
Daily life								
	Ages	M, 92	—	M, 92	*Alu*I, *Bsl*I, *Hae*III, *Msp*I	**&**	HA323	16, 20
	
	Over a hundred years old	F			*Bsl*I	**&**	B505	*$3*
	
	Residential areas	40 + 30 + 35 + 16	(4-NP)	121	*Hha*I, *Msp*I	**#**	Hh32	18
	
	BMI/obese, Lean	M, 92	—	M, 92	*Alu*I, *Bsl*I, *Hae*III, *Msp*I	**&**	A95	16, 22
		10 - 10	—	20	NGSA	**&**	*Clostridiales*	23
Disease								
	Smoking habit	M, 16	M,76	M, 92	*Alu*I, *Bsl*I, *Hae*III, *Msp*I	**&**	HA291	15, 16, 17, 21
	
	Nicotine-gum	10	10	20	*Bsl*I	**&**	B517	*$1*
	
	Smoking cessation period	M, 35	M, 57	M, 92	*Alu*I, *Bsl*I, *Hae*III, *Msp*I	**&**	M216	16
	
	Drinking habits	M, 45	M, 47	M, 92	*Alu*I, *Bsl*I, *Hae*III, *Msp*I	**&**	A47	16, 17
	
	Crohn’s disease	66 + 51 + 43 (3-NP)	121	281	*Hha*I, *Msp*I	**#**	Hh93	19
	
	Coronary artery disease	39	30	69	*Bsl*I	**#**	B853	24
	
	Sarcopenia, loss of grip/muscle mass	M			NGSA	**&**, **#**	*Odoribacteraceae*	*$3*
		F			NGSA	**#**	*Bacteroidaceae*	
	
	Sarcopenia (by Amino acid compositions *$2*)	M			21 kinds of amino acids	**&**	α-aminobutyric acid	*$3*
		F			21 kinds of amino acids	**#**	α-aminobutyric acid	
	
	Diabetes	M, 8 + 9 (2-NP)	M, 19	M, 36	*Bsl*I	**&**	B749	25
		F, 7 + 6 (2-NP)	F, 12	F, 25	*Bsl*I	**#**	B366	25
Technical aspects of DM-analyses								
	Restriction enzymes	Operating examples were Smoking, Drinking habits and Ages.			*Alu*I, *Bsl*I, *Hae*III, *Msp*I, *QAlu*I, *QHha*I, *QMsp*I	**&**, **#**	—	17, 21
	
	Prediction accuracies	Operating examples were Smoking and Drinking habits.			*Alu*I, *Bsl*I, *Hae*III, *Msp*I, *QAlu*I, *QHha*I, *QMsp*I	**&**, **#**	—	17, 22
		M, 92	—	M, 92				
	
	Personal identification	M, 92	—	M, 92	*Alu*I, *Bsl*I, *Hae*III, *Msp*I, *QAlu*I, *QHha*I, *QMsp*I	**&**	—	21

Restriction enzymes: *Bsl*I:516f-*Bsl*I, *Hae*III:516f-*Hae*III, *Msp*I:27f-*Msp*I, *Alu*I:27f-*Alu*I, *QHha*I:35f-*Hha*I, *QMsp*I:35f-*Msp*I and *QAlu*I:35f-*Alu*I; DM, data-mining; Ref, reference; NGSA, 16S rRNA sequencing amplicon analysis; M, male; F, female; NP, nominal partition. *$1*: same subjects, before and after the applying Nicotine-gum for a month, Akira Andoh, Shiga Univ. of Medical Science, personal communication. *$2*: instead of intestinal microbiota in feces, amino acid composition in the fasting blood collected early in the morning. *$3*: successful researches and analyses have been in progresses now. **&**: DM results were good, no error of classification in Decision-tree with 5 steps or less; **#**: few errors with 5 steps of Decision-tree. The grey cells are described in further detail.

**Table 3:** List of comparing prediction accuracies for specific examples

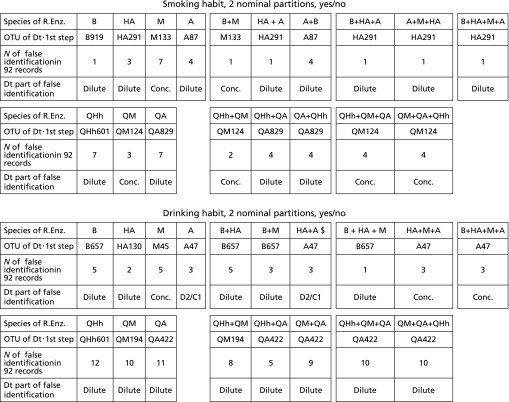

Evaluation of constructed Dt with 7 kinds of R.Enz and their combinations for smoking and drinking habits. The horizontal line of ‘*N* of false identifications in 92 records’ show the accuracies of identification. R.Enz, restriction enzyme; Dt, decision tree; *N*, number; B, *Bsl*I; HA, *Hae*III; M, *Msp*I; A, *Alu*I; QHh, 35f-*Hha*I; QM, 35f-*Msp*I; QA, 35f-*Alu*I; Dilute·Conc., location of false identified nodes in Dt; D2/C1·$, features of false nodes in Dt. Reprinted with permission from Ref (17).

## Abbreviations

CAD: coronary artery disease
DM: data mining analysis
Dt: decision tree of DM
IBD: inflammatory bowel diseases
NGSA: 16S rRNA sequencing amplicon analysis
NP: nominal partition of subjects at DM processing
OTU: operational taxonomic unit of T-RFLP
RE/R.Enz.: restriction enzyme of T-RFLP
T-RFLP: terminal restriction fragment length polymorphism
